# Repeated Foliar Glycine Betaine Applications in Cool-Season Greenhouse Passion Fruit: Effects of Application Frequency on Growth and Marketable Yield

**DOI:** 10.3390/plants15213455

**Published:** 2026-11-05

**Authors:** Yu-Xuan Tian, Syuan-You Lin

**Affiliations:** Department of Horticulture, National Chung Hsing University, No. 145, Xingda Road, South District, Taichung City 402202, Taiwan

**Keywords:** application frequency, plant biostimulant, *Passiflora edulis*, protected cultivation, sustainable crop production

## Abstract

Glycine betaine (GB) is used as an abiotic-stress biostimulant, but the performance of repeated foliar applications in perennial fruit crops under commercial conditions remains poorly defined. We evaluated four application strategies delivering cumulative GB inputs of 0, 42.5, 59.8, or 99.7 kg·ha^−1^ to greenhouse-grown ‘Tainung No. 1’ passion fruit (*Passiflora edulis* × *P*. *edulis* f. *flavicarpa*) during the cool season. Greenhouse temperature, vegetative growth, leaf greenness, visible reproductive development, marketable yield, and fruit quality were assessed. During the treatment period, 59 h below 10 °C were recorded, predominantly at night (83.1%). The 42.5 and 59.8 kg·ha^−1^ strategies did not consistently improve crop performance relative to the control. The 99.7 kg ha^−1^ strategy was associated with lower main-vine length during crop development, although final main-vine length at 144 DAT did not differ significantly from the control. The first visible flower bud appeared at a significantly higher node under 99.7 kg·ha^−1^ than in the control (27.1 vs. 20.8). Its appearance was significantly later under 99.7 than under 42.5 kg·ha^−1^ (29.9 vs. 19.6 DAT), but did not differ significantly from the control (21.0 DAT). The 99.7 kg ha^−1^ strategy also reduced marketable fruit number and yield by 14.6% and 13.8%, respectively. Fruit dimensions, individual fruit mass, and most quality traits were not consistently affected by GB, whereas harvest date more consistently influenced fruit quality. Under the recorded fluctuating thermal conditions, the intensive GB strategy was unfavorable, supporting crop-specific validation before repeated use.

## 1. Introduction

Passion fruit (*Passiflora* spp.) is a perennial vine produced widely in tropical and subtropical regions for fresh consumption and juice processing [1,2]. In Taiwan, the hybrid cultivar ‘Tainung No. 1’ (*P*. *edulis* × *P*. *edulis* f. *flavicarpa*) is commercially important because of its self-compatibility, high productivity, large fruit, and favorable flavor. Protected cultivation combined with supplemental night lighting enables fall establishment, winter growth, and spring fruit production [3,4]. This system extends the marketing season and reduces exposure to summer typhoons and heavy rainfall, but exposes vegetative and early reproductive development to cool-season temperatures that may restrict shoot growth, flowering, and fruit development [5].

Flowering in passion fruit is closely linked to continuous shoot growth. In ‘Tainung No. 1’, floral structures arise singly in the axils of newly formed leaves adjacent to tendrils, and floral primordia at basal nodes may fail to develop into externally visible flower buds until the shoot reaches sufficient developmental maturity and environmental conditions become favorable [6]. Accordingly, the node position of the first visible flower bud reflects not only node production but also the development and persistence of axillary reproductive structures. Controlled-environment studies demonstrate that temperature is a major determinant of both shoot growth and flower formation: ‘Tainung No. 1’ plants maintained at 20/15 °C produced shorter internodes, developed new nodes more slowly, and failed to form visible flower buds, whereas plants grown at 25/20 or 30/25 °C formed flower buds [7,8]. Plants at 25/20 °C formed their first visible flower buds at lower nodes and exhibited less flower-bud abortion than those at 30/25 °C [8]. Low-temperature inhibition of flowering at 20/15 °C was also associated with increased ethylene production, while inhibition of ethylene responses restored flower formation under the same temperature regime [8]. Photoperiodic regulation of flower development has also been demonstrated in passion fruit [9], providing a biological basis for supplemental night lighting in cool-season production. These findings support cool temperature as an important constraint on visible reproductive development for cool-season production of ‘Tainung No. 1’ passion fruit.

Low temperature disrupts membrane integrity, metabolism, photosynthetic processes, and developmental progression, with the severity of injury depending on both temperature intensity and exposure duration [10]. Plant biostimulants can be used to enhance crop performance, nutrient-use processes, product quality, or tolerance to abiotic stress [11]. Glycine betaine (GB), a compatible quaternary ammonium solute, can stabilize membranes and proteins, contribute to osmotic adjustment, and protect photosynthetic components under environmental stress [12,13]. GB has improved chilling responses in controlled studies with tomato and pepper seedlings [14,15], whereas the magnitude of the benefit depends on application concentration and stress context [16]. In passion fruit, a study using 20 and 40 mM GB reported location- and cultivar-dependent vegetative responses without a significant overall survival benefit under severe winter cold [17]; another study examined 100 mM GB responses to drought in yellow passion fruit [18].

However, results from short-term controlled-environment studies with vegetable seedlings cannot be assumed to predict the outcome of repeated applications in perennial fruit crops under commercial production conditions [14,15,19,20,21]. Existing passion fruit studies have focused mainly on GB-mediated responses to cold or drought [17,18], while the effects of cumulative seasonal exposure on reproductive development, yield, and fruit quality remain poorly defined. Foliar-applied GB can be absorbed by leaves and redistributed to other plant organs, indicating that repeated sprays may result in systemic exposure rather than remaining confined to the treated leaf surface [22]. Responses to exogenous GB are also concentration-dependent. In eggplant, 40 mM GB produced stronger growth and photosynthetic responses than 60 mM, with net photosynthetic rate declining at the higher concentration relative to the intermediate treatment [21]. In grapevine, exogenous GB was taken up and accumulated in plant tissues, while 50 mM reduced final leaf area and concentrations of 100 mM or greater severely inhibited growth [23].

We evaluated greenhouse-grown ‘Tainung No. 1’ passion fruit treated with a GB-free control or a common GB spray concentration applied at three frequencies (7, 10, and 19 sprays), resulting in cumulative active-ingredient inputs of 42.5, 59.8, and 99.7 kg·ha^−1^. We asked whether repeated GB application improved growth, visible reproductive development, marketable yield, or fruit quality under a naturally fluctuating cool-season environment, and whether the most intensive strategy provided additional benefit. We hypothesized that moderate application frequencies might support performance, whereas increasing application frequency and cumulative input would not necessarily yield further benefit and could be associated with unfavorable growth.

## 2. Results

### 2.1. Greenhouse Temperature and Low-Temperature Exposure

During the winter summary period (1 December 2025 to 28 February 2026), the mean daily maximum and minimum temperatures were 29.5 and 12.9 °C, respectively, resulting in a mean daily temperature range of 16.6 °C (Table 1). The lowest hourly observation during the GB treatment period was 7.0 °C on 4 January 2026 at 06:25 AM.

During the GB treatment period, the daily minimum temperature fell below 10 °C on 12 days. Cumulative exposure below 10 °C totaled 59 h, of which 10 h occurred during daytime and 49 h occurred during nighttime. Thus, 83.1% of the total exposure below 10 °C occurred at night, indicating that low-temperature events were predominantly nocturnal.

During the flowering observation period, the mean 24 h temperature was 18.6 °C. Mean daytime and nighttime temperatures were 22.7 and 15.0 °C, respectively. These results indicate that the plants experienced intermittent low-temperature episodes, particularly at night, whereas daytime temperatures generally remained substantially higher.

### 2.2. Vegetative Growth and Leaf SPAD

**Figure 1:**
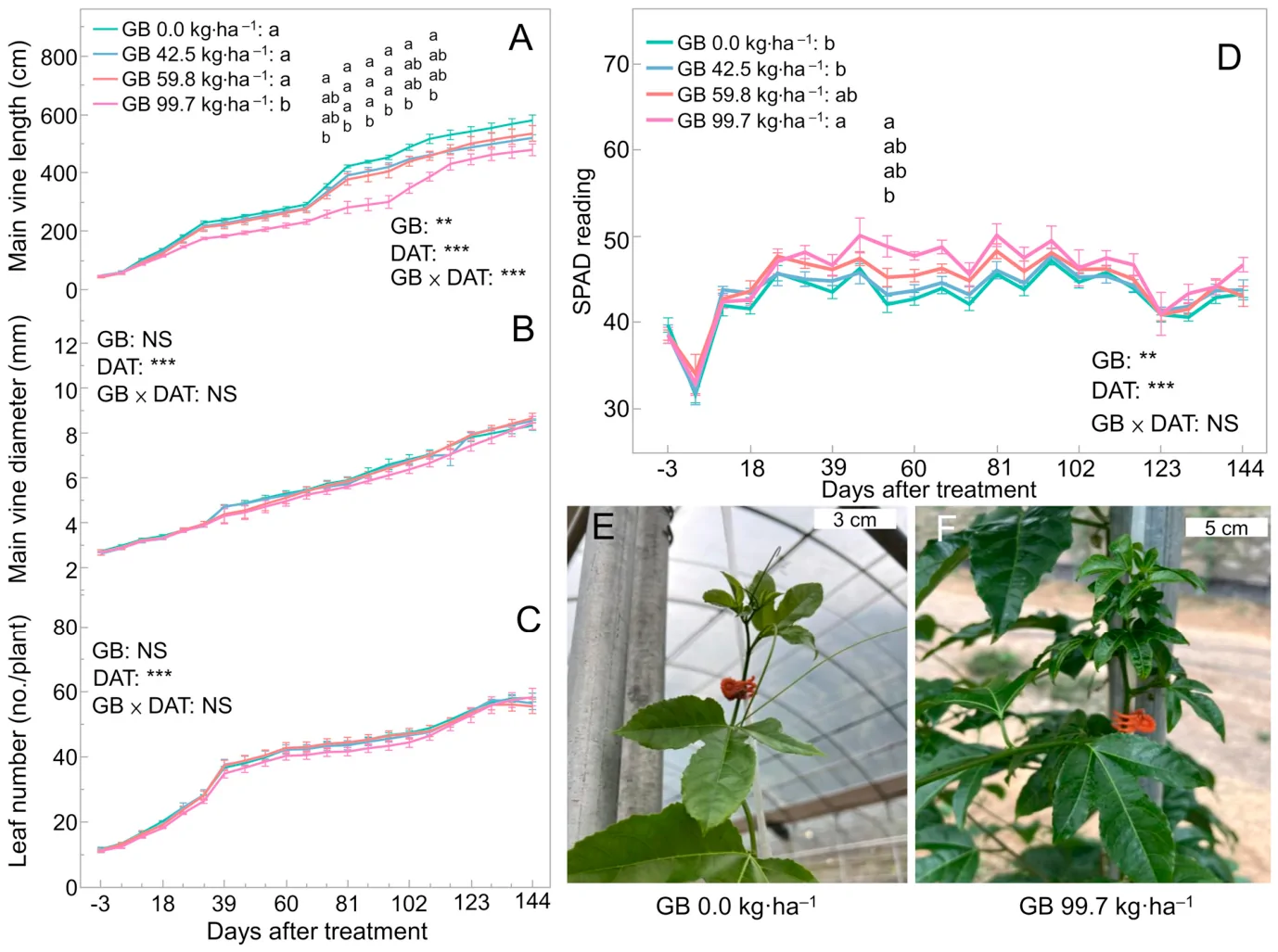
Effects of cumulative glycine betaine (GB) application strategies on vegetative growth, leaf SPAD readings, and shoot-tip morphology of ‘Tainung No. 1’ passion fruit. Main-vine length (**A**), main-vine diameter (**B**), number of leaves on the main vine (**C**), and SPAD readings of fully expanded main-vine leaves (**D**) were recorded from 3 days before treatment to 144 days after the first GB treatment (DAT). Representative shoot tips are shown for the control at 46 DAT (**E**) and the 99.7 kg·ha^−1^ treatment at 74 DAT (**F**). The control shoot showed normal internode elongation and leaf arrangement, whereas the 99.7 kg·ha^−1^ treatment showed shortened apical internodes, compact leaf arrangement, and clustered, morphologically abnormal young leaves. Photographs are presented as qualitative records and were not taken on the same date; therefore, they should not be interpreted as a direct time-matched comparison. Scale bars represent 3 cm in (**E**) and 5 cm in (**F**). Data in (**A**–**D**) are means ± SE (*n* = 5). GB, DAT, and GB × DAT denote the effects of cumulative GB application rate, days after treatment, and their interaction, respectively. NS, **, and *** indicate nonsignificant effects or significance at *p* < 0.01 and *p* < 0.001, respectively. Different lowercase letters adjacent to means indicate significant differences among GB treatments within a measurement date, whereas letters following treatment labels in the legend indicate significant differences among overall treatment means (Tukey’s HSD test, *p* < 0.05).

Cumulative GB application strategy, days after treatment (DAT), and their interaction significantly affected main-vine length (Figure 1A). At 32 DAT, the 0, 42.5, and 59.8 kg·ha^−1^ treatments had reached the 2 m horizontal trellis, whereas vines receiving 99.7 kg·ha^−1^ averaged 174.8 cm and reached the trellis at 53 DAT. At 144 DAT, main-vine length was 578.6, 519.5, 532.4, and 484.0 cm under 0, 42.5, 59.8, and 99.7 kg·ha^−1^, respectively. Although the mean under 99.7 kg·ha^−1^ was 16.3% lower than the control, the pairwise difference at 144 DAT was not significant (*p* = 1.000). Main-vine diameter and leaf number increased over time but were not significantly affected by GB strategy or its interaction with DAT (Figure 1B,C).

**Figure 2:**
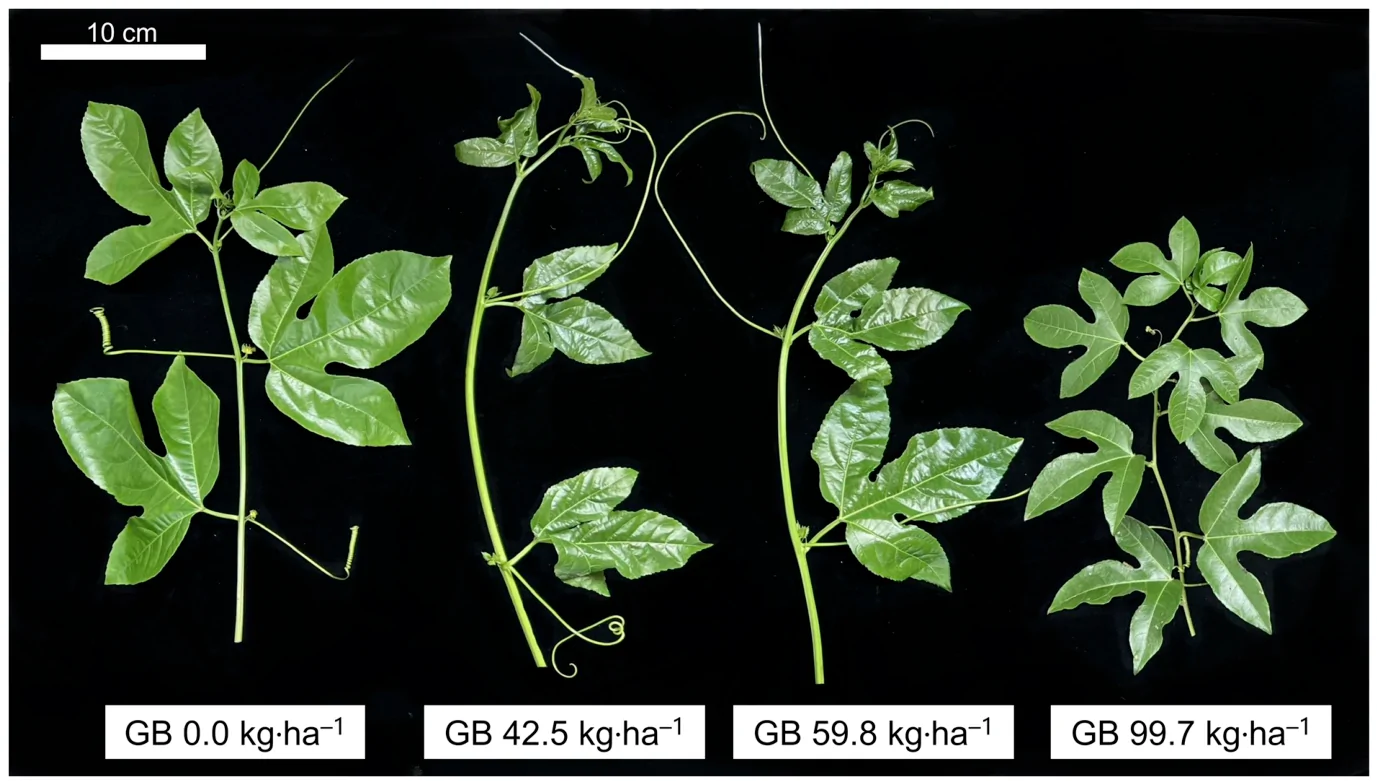
Representative shoot morphology of ‘Tainung No. 1’ passion fruit under different cumulative glycine betaine (GB) application rates. Shoots were collected at 133 d after treatment (DAT) from plants receiving cumulative GB application rates of 0, 42.5, 59.8, or 99.7 kg·ha^−1^, shown from left to right. Scale bar = 10 cm.

SPAD reading (a relative leaf-chlorophyll/greenness index) was affected by GB strategy and DAT, without a significant interaction (Figure 1D). Across measurement dates, the 99.7 kg·ha^−1^ treatment had higher SPAD values (45.3) than that of the control (42.8) and 42.5 kg·ha^−1^ treatments (43.3). Shortened apical internodes, compact leaf arrangement, and morphologically abnormal young leaves were also observed in the 99.7 kg·ha^−1^ treatment during the period of strongest growth suppression (Figure 1E and Figure 2).

### 2.3. Onset of Visible Reproductive Development

Cumulative GB application strategy significantly affected both the node position and DAT of the first visible flower bud, but not the timing of first flower opening (Table 2). The first visible flower bud occurred 6.3 nodes higher under the 99.7 kg ha^−1^ strategy than in the control. Its appearance at 29.9 DAT under 99.7 kg·ha^−1^ was numerically 8.9 d later than in the control (21.0 DAT); however, these two treatments did not differ significantly in Tukey’s HSD test. The first visible bud appeared significantly earlier under 42.5 kg·ha^−1^ (19.6 DAT) than under 99.7 kg ha^−1^. First flower opening ranged from 56.5 to 62.6 DAT without a significant treatment effect.

**Figure 3:**
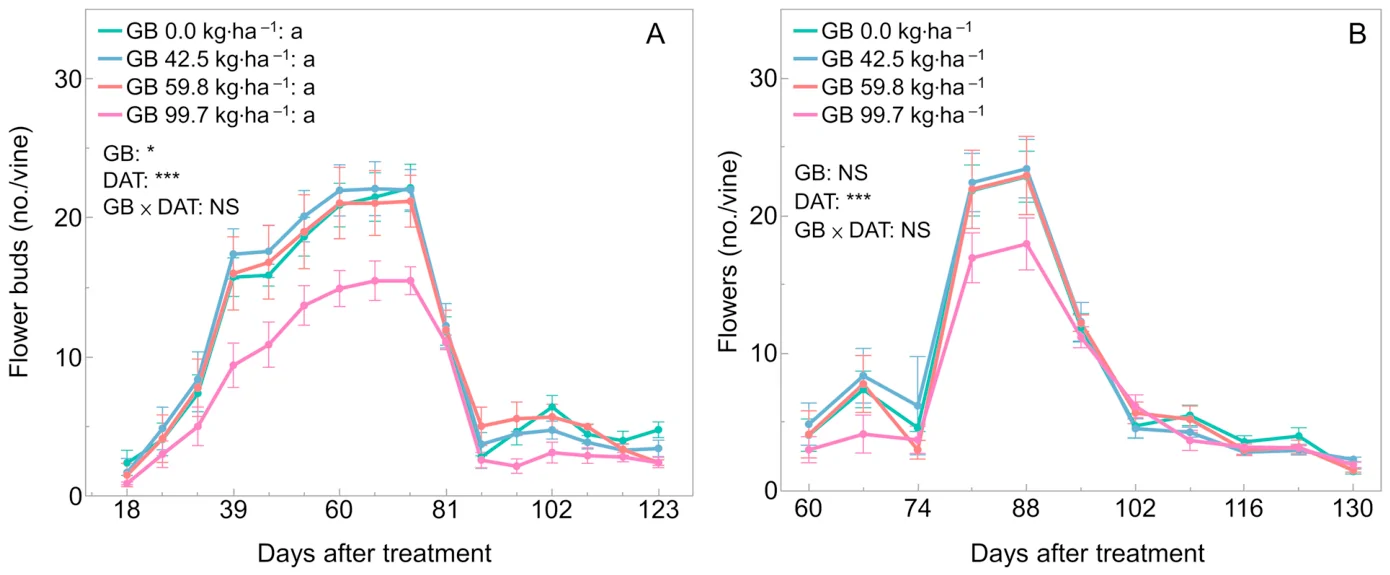
Effects of cumulative glycine betaine (GB) application strategy on the number of flower buds (**A**) and flowers (**B**) on the main vine in ‘Tainung No. 1’ passion fruit. GB, DAT, and GB × DAT indicate the effects of glycine betaine treatment, days after treatment, and their interaction, respectively. NS indicates not significant; * and *** indicate significance at *p* < 0.05 and *p* < 0.001, respectively. Data are means ± SE (*n* = 5). Same lowercase letters or no letter in the legend or at a measurement date indicate non-significant differences (Tukey’s HSD test, *p* < 0.05).

Flower bud number on the main vine was significantly affected by GB strategy (*p* = 0.032) and DAT, without a significant interaction (Figure 3A); however, Tukey’s HSD test did not detect significant pairwise differences among the GB treatments. Numerically, flower bud number increased from 18 DAT and peaked from approximately 60 to 81 DAT. During this period, 0, 42.5, and 59.8 kg·ha^−1^ treatments generally had 21–22 buds per plant, whereas the 99.7 kg·ha^−1^ treatment had approximately 15–16. Flower number on the main vine was affected by DAT but not by GB strategy or the GB × DAT interaction (Figure 3B). The numerically lower flower number under 99.7 kg ha^−1^ therefore represents a trend rather than a statistically supported treatment effect.

### 2.4. Marketable Fruit Number and Marketable Yield

**Figure 4:**
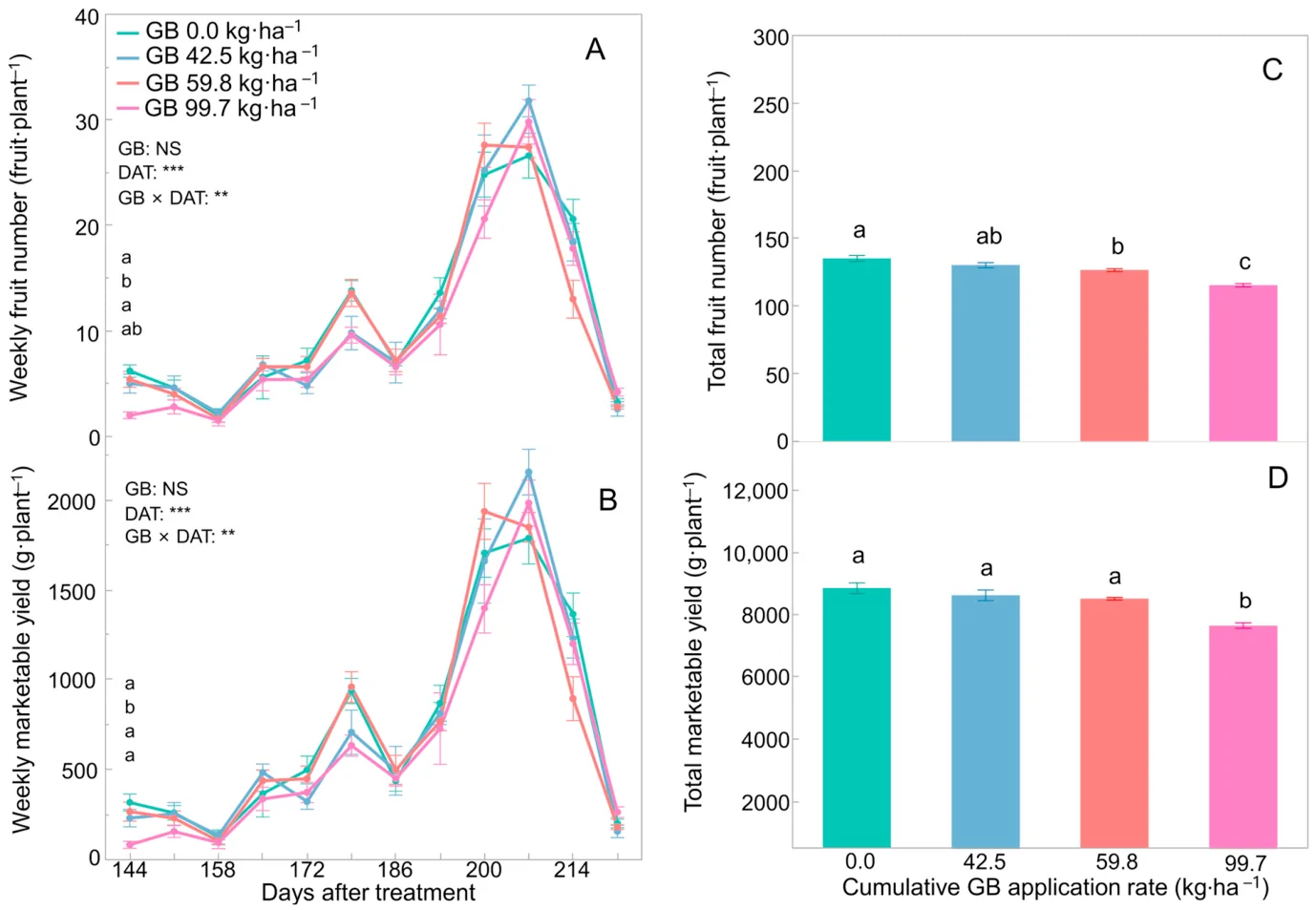
Effects of cumulative glycine betaine (GB) application strategy on weekly marketable fruit number (**A**), weekly marketable yield (**B**), total marketable fruit number (**C**), and total marketable yield (**D**) in ‘Tainung No. 1’ passion fruit. In panel (**A**) and (**B**), GB, DAT, and GB × DAT indicate the effects of glycine betaine treatment, days after treatment, and their interaction, respectively. NS indicates not significant; ** and *** indicate significance at *p* < 0.01 and *p* < 0.001, respectively. Data are means ± SE (*n* = 5). Same lowercase letters or no letter in the legend or at a measurement date indicate non-significant differences (Tukey’s HSD test, *p* < 0.05).

Weekly marketable fruit number and weekly marketable yield were not significantly affected by the overall GB main effect, but both traits were strongly affected by harvest date and showed significant GB × DAT interactions (Figure 4A,B). At the first harvest (144 DAT), marketable fruit number was 6.2, 5.0, 5.4, and 2.0 fruit·plant^−1^ under 0, 42.5, 59.8, and 99.7 kg·ha^−1^, respectively. The control and 59.8 kg·ha^−1^ treatments had significantly more marketable fruit than the 99.7 kg·ha^−1^ treatment, whereas 42.5 kg·ha^−1^ was intermediate. The weekly marketable yield at 144 DAT was 316.7, 230.4, 266.4, and 83.1 g·plant^−1^, respectively, with the 99.7 kg·ha^−1^ treatment significantly lower than the other three treatments. Thereafter, treatment rankings varied among harvest dates. The first harvest peak occurred near 179 DAT, followed by a larger peak near 207 DAT. At 207 DAT, the 42.5 kg·ha^−1^ treatment reached 31.7 fruit·plant^−1^ and 2141.0 g·plant^−1^, but the crossover among treatments across harvest dates prevented a consistent overall advantage of any GB strategy.

Total marketable fruit number declined with increasing GB input (Figure 4C). Values were 135.1, 130.1, 126.6, and 115.4 fruits plant^−1^ under 0, 42.5, 59.8, and 99.7 kg ha^−1^, respectively. The control did not differ from 42.5 kg·ha^−1^ but exceeded 59.8 and 99.7 kg·ha^−1^; the highest cumulative input was lower than all other treatments. Total yield was 8852.6, 8626.6, 8524.3, and 7631.4 g·plant^−1^, respectively (Figure 4D). The 99.7 kg·ha^−1^ treatment reduced total marketable yield by 13.8% relative to the control and was significantly lower than the other three treatments, which did not differ from one another.

### 2.5. Fruit Quality

GB strategy did not significantly affect fruit length, diameter, mass, or the overall main effects for peel L*, a*, and b* values (Table 3). Harvest date significantly affected fruit mass, L*, and b*, and the GB × DAT interaction was significant only for L*. Individual fruit mass increased from 56.6 g at 187 DAT to 63.4 g at 194 DAT and 71.2 g at 200 DAT. Over the same period, peel L* declined from 30.2 to approximately 25.0, and b* declined from 8.9 to approximately 5.4, indicating darker and less-yellow peel at later harvests.

GB strategy did not significantly affect the overall main effects for soluble solids content (SSC), titratable acidity (TA), or juice content, although it affected the SSC/TA ratio and interacted with harvest date for SSC and SSC/TA (Table 4). For SSC, treatment differences varied among harvest dates and did not show a consistent response to cumulative GB application. TA decreased from 2.58% at 187 DAT to 2.08% at 200 DAT, while SSC/TA increased from 7.39 to 8.93. Juice content increased from 41.3% at 187 DAT to 42.3% at 194 DAT and 43.3% at 200 DAT; however, Tukey’s HSD test detected no significant pairwise differences among sampling dates. Overall, harvest timing had a stronger and more consistent influence on fruit physical and compositional traits than cumulative GB application.

## 3. Discussion

### 3.1. Flowering Under Fluctuating Cool-Season Temperatures

The thermal environment provides an essential context for interpreting the limited benefit of GB in this study. Controlled-environment studies with ‘Tainung No. 1’ have demonstrated substantial suppression of vegetative and reproductive development under sustained cool conditions. Plants maintained at 20/15 °C showed slower shoot growth and node production and failed to form visible flower buds, whereas plants grown at 25/20 or 30/25 °C flowered [7,8]. Inhibition under 20/15 °C was associated with increased ethylene production [8]. More severe cold exposure (5–10 °C) can also restrict shoot growth and subsequent fruit development in passion fruit [5].

The greenhouse thermal regime in the present study was not directly comparable with the constant day/night temperature treatments used in previous controlled-environment experiments. During the flowering observation period, mean daytime and nighttime temperatures were 22.7 and 15.0 °C, respectively. Thus, the mean nighttime temperature was identical to the 15 °C night temperature used in the 20/15 °C regime that suppressed flowering in ‘Tainung No. 1’ [8], whereas daytime temperatures in the greenhouse were generally higher and fluctuated substantially over time. In addition, although 59 h below 10 °C were recorded during the GB treatment period, these events were intermittent and occurred predominantly at night. The <10 °C measurements therefore characterize the frequency and duration of the coldest episodes rather than an established biological threshold for flower inhibition.

Despite intermittent nocturnal cooling, control plants continued vine growth, developed visible flower buds, flowered, and produced marketable fruit. These observations demonstrate that reproductive development occurred under the recorded greenhouse conditions, but they do not establish that the thermal regime was non-limiting or optimal. The absence of a warm reference treatment prevents direct estimation of the degree of cold-related growth or reproductive suppression. Accordingly, the lack of benefit from 42.5 and 59.8 kg·ha^−1^ should be interpreted as a lack of measurable GB advantage under the fluctuating cool-season conditions encountered here, rather than as evidence that the crop was unaffected by low temperature.

### 3.2. Growth Responses to Intensive GB Application and Formulation-Related Confounding

At the fixed foliar concentration tested, the highest-input GB strategy was associated with reduced vine elongation, altered shoot-tip morphology, and lower marketable productivity, whereas less frequent applications did not consistently improve performance. The solution contained 11.7 g·L^−1^ GB active ingredient (~100 mM), above the 20 and 40 mM concentrations tested in the Lebanese passion fruit trial [17]. In eggplant, 40 mM GB produced more favorable physiological responses than 60 mM [21]. However, these cross-species comparisons do not establish a critical concentration for passion fruit, particularly because all GB treatments in the present study used the same solution concentration.

At a broader scale, a field-trial meta-analysis across multiple crops and non-microbial biostimulant categories found that yield benefits increased only slightly with up to four applications, whereas more frequent applications were associated with lower yield responses [20]. This broad relationship provides context but cannot identify the cause of the present response. The 42.5, 59.8, and 99.7 kg·ha^−1^ strategies resulted from 7, 10, and 19 GB applications, respectively; cumulative active-ingredient input was therefore confounded with application frequency, timing, and the plant stage at each spray. The highest-input response cannot be attributed to cumulative GB dose alone.

An important control feature is that the GB-free treatment received water plus 0.02% surfactant during 19 spraying events, matching the number of events in the 99.7 kg·ha^−1^ strategy. Consequently, frequency of handling and wetting events alone is unlikely to explain the difference between these two strategies. Nevertheless, the treatment solutions differed in GB formulation chemistry, canopy-dependent spray volumes could differ between strategies as plants developed, and the experiment did not independently manipulate surfactant amount, formulation constituents, or spray concentration. Physical spray effects and chemical interactions therefore cannot be fully ruled out. The observed result should be attributed to the tested commercial application strategy, not to proven GB-specific phytotoxicity.

Leaf SPAD readings were higher under the 99.7 kg·ha^−1^ strategy but were not accompanied by greater vine growth or yield. SPAD is influenced by chlorophyll concentration, leaf nitrogen (N) status, anatomy, and leaf thickness and cannot be equated directly with photosynthetic capacity [24,25]. The formulation reported 11% total N by product mass (as shown in Section 4.3), but N is also an intrinsic constituent of the GB molecule; the N associated with the product should not automatically be treated as independently supplied mineral fertilizer N. Because no nutrient-N-matched treatment or tissue-N analysis was included, the possible nutritional contribution of absorbed or metabolized formulation N cannot be evaluated. The shoot abnormalities are consistent with an unfavorable response, but measurements needed to establish phytotoxicity or its mechanism (e.g., tissue GB, electrolyte leakage, osmotic and oxidative markers) were not obtained.

### 3.3. The Highest-Input Strategy Altered the Onset of Visible Reproductive Development

Flower-bud appearance was assessed as a visible morphological endpoint, not as the timing of histological floral initiation. In ‘Tainung No. 1’, reproductive structures arise in leaf axils of newly formed nodes, but early primordia may not immediately develop into visible flower buds [6]. Thus, first-bud position and timing combine node development, progression of floral primordia, and persistence of axillary structures.

The first visible flower bud appeared at node 27.1 and 29.9 DAT under 99.7 kg·ha^−1^, compared with node 20.8 and 21.0 DAT in the control. The 6.3-node difference was significant, whereas the numerical 8.9 d timing difference from the control was not significant in the post hoc comparison; the significant timing contrast was between 42.5 and 99.7 kg·ha^−1^ (Table 2). These observations support an alteration in the position and timing of visible reproductive development, but not a demonstrated delay in floral initiation itself.

The higher first-bud node coincided with slower vine extension and altered shoot morphology under the highest-input strategy. Retained flower-bud counts showed an overall GB effect, but treatment pairs did not differ by Tukey’s HSD test, and fully open flower counts were not significantly affected by GB. Therefore, a sustained reduction in flower production or a causal path from visible first-bud position to marketable yield cannot be established. Node-level anatomical observations and tracking of individual reproductive structures would be required to identify the affected developmental process.

### 3.4. Lower Marketable Yield Was Associated with Fewer Marketable Fruits

Total marketable fruit number was 135.1 fruit·plant^−1^ in the control and 115.4 fruit·plant^−1^ under 99.7 kg·ha^−1^, while marketable yield was 13.8% lower under the highest-input strategy. Individual fruit fresh mass and fruit dimensions did not significantly differ among GB strategies. Thus, the lower marketable yield was associated primarily with fewer marketable fruits rather than lower individual fruit mass.

Weekly bud and flower counts did not demonstrate a persistent significant reduction in flower number under the high-input strategy. Moreover, only marketable fruits were counted and weighed. Although the grading system does not impose a minimum fruit-mass threshold for marketability, fruits may still be excluded because of maturity, external defects, disease or insect damage, cracking, or other quality-related criteria (Table S1). The present measurements therefore cannot distinguish lower fruit set or retention from a lower proportion of harvested fruits meeting marketability criteria. Accordingly, the experiment does not establish that delayed visible-bud development caused the lower marketable fruit number.

The practical conclusion is that the intensive strategy reduced marketable output under the observed conditions. Future work should track individual flowers and developing fruits through fruit set, retention, harvest, and grading, while ensuring unequivocal assignment of harvested fruits to experimental plots. Such records would help determine which reproductive or quality-control step accounted for the lower marketable fruit number.

### 3.5. Harvest Date More Consistently Influenced Fruit Quality than GB Strategy

Across the three sampling dates, individual fruit fresh mass increased while peel CIE L* and b* declined; TA decreased and the SSC/TA ratio increased. Similar physical and compositional changes have been reported during passion fruit maturation [26,27,28,29]. Because sampling was confined to 187, 194, and 200 DAT, these patterns represent the sampled fruit rather than necessarily all fruits collected throughout the 12-harvest season.

GB strategy had no consistent main effect on fruit dimensions, fruit mass, SSC, TA, or the juice content. Some significant interactions with harvest date were not accompanied by a stable improvement across dates. Thus, the reduction in marketable yield under the most intensive GB strategy was not offset by a consistent quality benefit.

### 3.6. Implications for Sustainable Biostimulant Use

This study evaluated repeated field application of a GB formulation rather than the mechanism of GB-induced chilling tolerance. Field-trial evidence shows that biostimulant responses vary with crop, environment, and application program [20], and similar seasonal and cultivar dependence has been reported for GB-containing treatments in sweet cherry (*Prunus avium* L.) [30]. In the present study, 7 or 10 GB applications (the treatments of 42.5 and 59.8 kg·ha^−1^, respectively) provided no consistent benefit, whereas 19 applications (the treatment of 99.7 kg·ha^−1^) at approximately 100 mM reduced marketable production.

These findings apply to one cultivar, greenhouse, and season. They do not exclude beneficial effects under more severe chilling, in other cultivars, or at different GB concentrations. Because concentration, application frequency, formulation-associated N, and exposure duration were not separated experimentally, the cause of the high-input response cannot be identified.

Future studies should independently test GB concentration and application frequency, include appropriate controls for formulation-associated N, and compare defined thermal regimes including a warm reference. Physiological measurements should also be synchronized with verified cold events. Until such validation is available, repeated calendar-based application of approximately 100 mM GB cannot be recommended for cool-season passion fruit production.

## 4. Materials and Methods

### 4.1. Experimental Site and Plant Materials

The experiment was conducted in a commercial passion fruit greenhouse in Zhongliao Township, Nantou County, Taiwan (23°53′ N, 120°44′ E; 123 m above sea level). Virus-free grafted plants of ‘Tainung No. 1’ passion fruit (*Passiflora edulis* × *P*. *edulis* f. *flavicarpa*) grafted onto seedling rootstocks of yellow passion fruit (*P*. *edulis* f. *flavicarpa*) were obtained from a commercial nursery on 17 October 2025. At receipt, plants were approximately 30–45 cm tall and had 10–14 nodes. They were planted in the ground on 20 October 2025.

The polyethylene-covered greenhouse was 60 m long, 18 m wide, and 10 m high, with 32-mesh insect netting on the sidewalls to a height of 3 m. Plants were trained to a horizontal trellis 2 m above the soil at a spacing of 6 m between rows and 1 m within rows (1667 plants·ha^−1^), with rows oriented north–south. One vertical support string was installed per plant. One main vine was retained and trained to the trellis. Consistent with the commercial grower’s standard practice, the main vine was not pinched after reaching the trellis. Six to seven lateral vines were selected as principal fruiting shoots; additional laterals were not removed. Fruit load was not artificially thinned, and naturally retained fruit were allowed to develop to harvest. These management practices were applied uniformly to all plots.

### 4.2. Supplemental Lighting and Management Practices

To support reproductive development during the naturally short photoperiod, supplemental night lighting was applied from planting until 20 February 2026. Full-spectrum 11 W LED lamps (ST-L011-W; Qi Meng, Taipei, Taiwan; nominal spectral range 400–720 nm; luminous flux 1600 lm) were positioned approximately 0.5 m above the trellis at 1 m spacing and operated daily from 18:00 to 22:00. Photosynthetic photon flux density (PPFD) at canopy height was not measured; therefore, the supplemental light intensity cannot be quantified. Supplemental lighting was discontinued on 20 February 2026 because the crop had entered the late flowering stage, consistent with the cooperating grower’s seasonal management practice. Flowering observations nevertheless continued until 9 March 2026.

Rice husk was incorporated into the planting strip before planting. An arbuscular mycorrhizal inoculant containing *Funneliformis mosseae* (syn. *Glomus mosseae*) was placed around each root ball at approximately 2.5 g·plant^−1^. Plants were manually irrigated with local stream water every two days.

Fertilization was adjusted according to crop developmental stage and applied uniformly to all experimental plots. During vegetative growth, a 15–15–15 N–P_2_O_5_–K_2_O soluble fertilizer (YaraRega™, Yara International ASA, Oslo, Norway) was applied as a soil drench at a 1:500 dilution, using 5166 L·ha^−1^ of diluted fertilizer solution per application. Two applications were made at four-week intervals. During flowering, a 3–4–25 N–P_2_O_5_–K_2_O fertilizer (YaraRega™, Yara International ASA, Oslo, Norway) was applied in the same manner at 5166 L·ha^−1^ per application, with two applications at four-week intervals.

Beginning approximately one month after flowering and continuing through fruit development, the 3–4–25 fertilizer and a high-K soluble fertilizer containing 12–12–36–2 N–P_2_O_5_–K_2_O–MgO, 0.02% B, and 0.002% Mo (Speedfol^®^, Sociedad Quimica y Minera de Chile S.A., Santiago, Chile) were alternated. Each product was applied at 10 kg·ha^−1^ per application, with three applications of each fertilizer during this period.

Calcium, magnesium, and micronutrients were supplied uniformly by foliar application every two weeks. The foliar fertilizers included YaraTera™ calcium fertilizer (14.4–0–0–26.3 N–P_2_O_5_–K_2_O–Ca; Yara International ASA, Oslo, Norway) and Haifa magnesium nitrate (10.5–0–0–15.5 N–P_2_O_5_–K_2_O–MgO; Haifa Group, Haifa, Israel). These materials were applied at a 1:1000 dilution using 3093 L·ha^−1^ of spray solution per application, for a total of 10 foliar applications. Crop-protection and other cultural practices were applied uniformly across all treatments.

For mite control, spiromesifen and chlorfenapyr were each applied twice at a 1:2000 dilution, with the two active ingredients used alternately for a total of four applications. All crop-protection applications were completed before flowering. No pesticides were applied after the onset of flowering.

### 4.3. Glycine Betaine Treatments and Experimental Design

**Figure A2:**
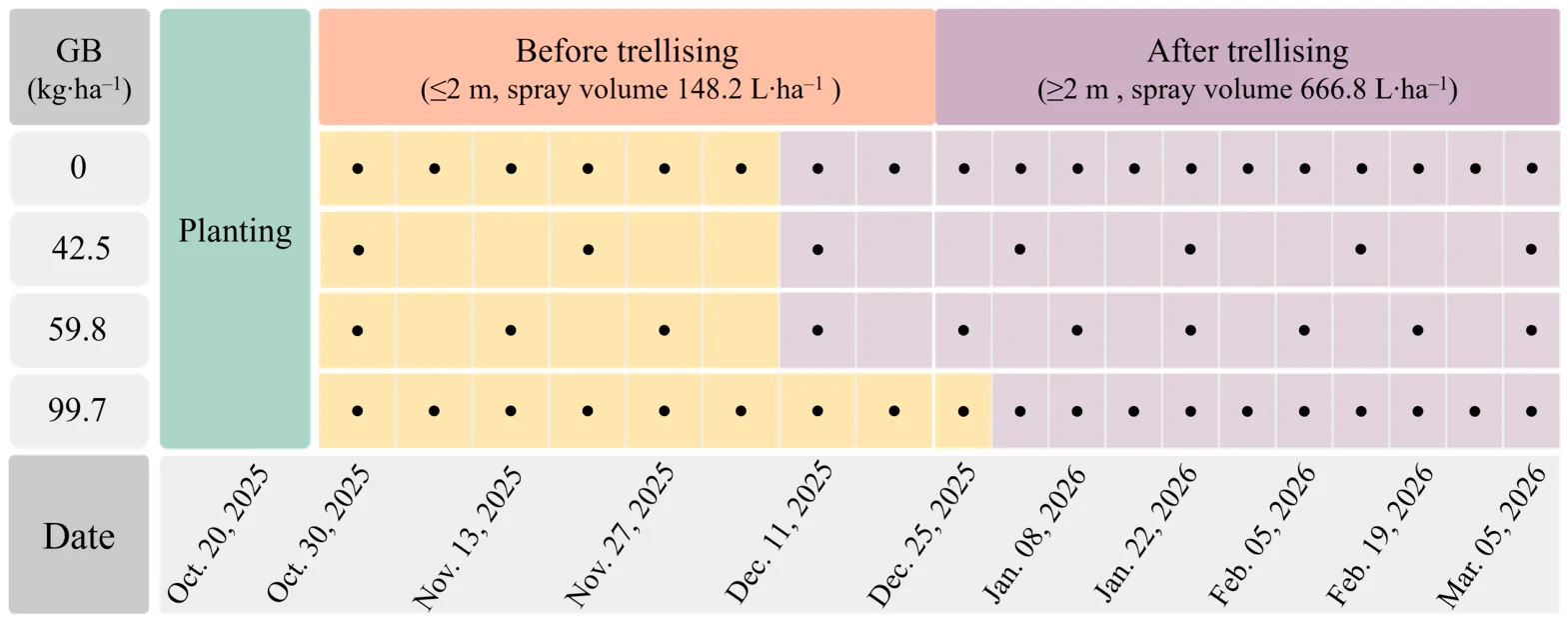
Application schedule and canopy-dependent spray volumes of glycine betaine (GB) in greenhouse-grown ‘Tainung No. 1’ passion fruit. Rows represent cumulative GB application rates (0, 42.5, 59.8, and 99.7 kg ha^−1^), and columns represent weekly application dates from 30 October 2025 to 5 March 2026. Black dots indicate individual spraying events.

The commercial formulation Greenstim^®^ (Phytochrome Inc., Tokyo, Japan) contained 97% glycine betaine (GB) and 11% total N by product mass. Spray solutions were prepared with 11.7 g·L^−1^ GB active ingredient (approximately 100 mM) and 0.02% (*v*/*v*) nonionic surfactant (Nian-Di; Sinon Corporation, Taichung, Taiwan). Four strategies resulted in cumulative GB inputs of 0, 42.5, 59.8, and 99.7 kg·ha^−1^ (Table 5). Applications occurred from 30 October 2025 to 5 March 2026 (Figure A2). Sprays were applied using a 20 L pressure sprayer operated at a flow rate of 26.67 mL·s^−1^. During application, the spray was directed to the abaxial (lower) leaf surface; the adaxial surface was not intentionally sprayed. Cumulative input was determined by the common solution concentration, canopy-dependent spray volume, and actual number of applications. Before vines reached the trellis (≤2 m), each spray delivered 148.2 L·ha^−1^ (approximately 0.089 L·plant^−1^). After trellising and lateral-vine development, spray volume increased to 666.8 L·ha^−1^ (approximately 0.400 L·plant^−1^). The 42.5, 59.8, and 99.7 kg·ha^−1^ strategies received 7, 10, and 19 GB applications, respectively. The control received water and surfactant during 19 control application events.

Treatments were arranged in a randomized complete block design (RCBD) with five blocks. Each block contained one plot of each GB strategy, and each plot consisted of three adjacent plants. The greenhouse contained 249 plants; 60 plants were designated for data collection and the remaining plants served as guards. The experimental unit was the three-plant plot. Plant- and fruit-level subsamples were averaged within plot before statistical analysis, providing five independent replicates per treatment.

### 4.4. Greenhouse Temperature Monitoring and Calculation of Low-Temperature Exposure

Greenhouse air temperature and relative humidity were recorded from 20 October 2025 to 15 April 2026 using a HOBO MX2301A temperature and relative humidity data logger (Onset Computer Corporation, Bourne, MA, USA). The logger was positioned near the center of the greenhouse, approximately 2.5 m above ground level, and shielded from direct solar radiation. Measurements were recorded at 1 h intervals.

Daily mean, maximum, and minimum temperatures were calculated from all hourly observations within each calendar day. The daily temperature range was calculated as the difference between the daily maximum and minimum temperatures. Mean daily maximum temperature, mean daily minimum temperature, and mean daily temperature range were subsequently calculated for the winter period from 1 December 2025 to 28 February 2026.

Low-temperature exposure was quantified for the GB treatment period, which extended from 30 October 2025 to 5 March 2026. The 10 °C threshold was used to characterize acute low-temperature exposure and was not assumed to represent a critical threshold for flower inhibition. Previous controlled-environment studies with ‘Tainung No. 1’ evaluated flowering under sustained day/night temperature regimes, including 20/15 °C, rather than short-term hourly exposure below a single threshold [8]. A day with a minimum temperature below 10 °C was defined as a calendar day containing at least one hourly temperature observation below 10 °C. Cumulative exposure below 10 °C was calculated by counting the hourly observations below this threshold, with each observation representing 1 h of exposure. The minimum recorded temperature and its corresponding date and time were also identified.

Greenhouse temperature data were summarized for three predefined observational windows: the GB treatment period (30 October 2025 to 5 March 2026), the winter summary period (1 December 2025 to 28 February 2026), and the flowering observation period (56.5–130 DAT; 25 December 2025 to 9 March 2026). Daytime and nighttime observations were classified according to the date-specific local sunrise and sunset times at the experimental site (23°53′ N, 120°44′ E), with daytime defined as sunrise to sunset and nighttime as sunset to sunrise on the following day. For the GB treatment period, cumulative exposure below 10 °C was calculated separately for daytime and nighttime, and the proportion of total low-temperature exposure occurring at night was determined. For the flowering observation period, mean 24 h, daytime, and nighttime temperatures were calculated. The winter summary period was used to characterize seasonal daily temperature conditions.

### 4.5. Vegetative Growth and SPAD Measurements

Baseline vegetative measurements were collected on 27 October 2025, 3 days before GB treatment. The graft union was ~10 cm above the soil surface. Main-vine length was measured weekly from the graft union to the shoot apex with a measuring tape. Node position was counted sequentially from the basal end of the scion, with the first node above the graft union designated as node 1; nodes already present on the nursery plant at transplanting were included in the count. Main-vine diameter was measured weekly with a digital caliper 2 cm above the tenth node. Fully expanded leaves on the main vine were counted weekly, whereas incompletely expanded leaves were excluded.

Relative leaf greenness was measured with a SPAD-502 meter (Spectrum Technologies, Aurora, IL, USA). For each plant, three fully expanded mature leaves between the third and sixth nodes below the apex of the main vine were selected. Two readings were taken per leaf and averaged, after which plant means were averaged within the plot.

### 4.6. Reproductive Development, Harvest, and Marketable Yield

Reproductive development on the main vine was assessed weekly. A flower bud was operationally defined as an unopened axillary flower bud at least 1 mm in length. A flower was considered fully open when the petals and corona were expanded and the anthers and stigmas were exposed. For each plant, the days after treatment (DAT) and node position of the first visible flower bud, as well as the DAT of the first fully open flower, were recorded.

At each weekly assessment, retained visible flower buds and fully open flowers remaining on the main vine were counted separately. A visible flower bud was defined as a clearly distinguishable reproductive bud emerging from the leaf axil, whereas a fully open flower was defined as a flower at anthesis with the floral organs fully expanded. The counts represented the reproductive structures present at the time of each assessment rather than newly formed structures since the preceding assessment or cumulative production over time. Abscised, senesced, or visibly aborted flower buds and flowers were excluded. A hive of honey bees (*Apis mellifera* L.) was placed in the greenhouse from 10 December 2025 to 1 March 2026 to facilitate pollination.

Twelve harvests were conducted at approximately 7-day intervals from 144 to 221 DAT, corresponding to 23 March through 8 June 2026. Catch nets were physically partitioned into nonoverlapping sections corresponding to each three-plant experimental plot. At each harvest, fruit were classified as marketable or nonmarketable according to the grading standards for improved passion fruit established by Taipei Agricultural Products Marketing Co., Ltd. (Taipei, Taiwan) [31]. Fruits meeting at least the minimum commercial-quality grade were considered marketable, whereas fruit with decay, cracking, disease or insect damage, physiological disorders, mechanical injury, or other defects that substantially affected appearance or quality were classified as unmarketable (Table S1). Only marketable fruit were counted and weighed. Weekly marketable fruit number and marketable yield were determined for each plant and expressed as fruit·plant^−1^ and g·plant^−1^, respectively. Total marketable fruit number and total marketable yield were calculated by summing the weekly values across the 12 harvests.

### 4.7. Fruit Quality Measurements

At three harvest dates (187, 194, and 200 DAT), fruits were held for two days after collection to simulate commercial postharvest conditioning. Six marketable fruits were randomly selected from the pooled harvest of the three plants within each experimental unit. Fruit dimensions (longitudinal and equatorial diameters) were measured with a digital caliper, and individual fruit mass (g·fruit^−1^) was measured with an electronic balance (JET3002G/00; Mettler Toledo, Greifensee, Switzerland).

Peel color was measured at the center of each of the three carpels with a ColorFlex EZ colorimeter (HunterLab, Reston, VA, USA), and the three readings were averaged per fruit. Color was expressed as CIE L*, a*, and b*. L*, a*, and b* are CIE color coordinates, where L* represents lightness, a* represents the green (−) to red (+) axis, and b* represents the blue (−) to yellow (+) axis. Juice was separated from the arils by pressing through a filter bag. SSC was measured with a PAL-1 digital refractometer (Atago, Tokyo, Japan) and expressed as °Brix. For TA, a 0.5 mL aliquot of juice was diluted with 29.5 mL of distilled water and titrated potentiometrically with 0.1 N NaOH to pH 8.0 using an automatic acidity titrator (TA-70; DKK-TOA, Tokyo, Japan). The instrument has a minimum dispensing volume of 0.01 mL and a burette repeatability of ±0.01 mL. The pH 8.0 endpoint was used consistently for all samples; results were expressed as % citric acid equivalents. SSC/TA was calculated. Juice content was determined gravimetrically from the fresh aril-plus-juice fraction and its dry mass after drying at 70 °C for 3 days [Equation (1)].

(1) $$\mathrm{Juice}\;\mathrm{content}\;\left(\%\right)=\frac{\left(\mathrm{fresh}\;\mathrm{aril}+\mathrm{juice}\;\mathrm{mass}-\mathrm{dry}\;\mathrm{mass}\right)}{\mathrm{whole}-\mathrm{fruit}\;\mathrm{fresh}\;\mathrm{mass}\;}\times 100$$

### 4.8. Statistical Analysis

Data were analyzed with generalized linear mixed models in JMP^®^ Student Edition 19 (SAS Institute Inc., Cary, NC, USA). The plot was the experimental unit. For repeated measurements, GB strategy, DAT, and GB × DAT were fixed effects, block was a random effect, and plot within block was specified as the subject for repeated observations. For variables measured once or summarized over the season, GB strategy was fixed and block was random.

Positive continuous responses, including vine length, vine diameter, fruit dimensions, fruit mass, yield, and SSC/TA, were modeled using lognormal distributions. Count responses, including leaf, flower bud, flower, weekly fruit, and cumulative fruit numbers, and integer phenology responses were modeled with Poisson distributions and log links. SPAD and peel-color coordinates were analyzed with Gaussian errors. TA and juice content were converted to proportions and analyzed using beta distributions with logit links; SSC was scaled to a 0–1 interval before beta modeling. Model adequacy was evaluated using studentized residual plots and other residual diagnostics. Multiple comparisons were conducted with Tukey’s HSD test, with statistical significance declared at *p* < 0.05.

## 5. Conclusions

Under the intermittent cool-season conditions encountered in this subtropical greenhouse, cumulative foliar GB inputs of 42.5 and 59.8 kg·ha^−1^ did not consistently improve passion fruit growth, reproductive development, yield, or fruit quality relative to the control. The highest cumulative input, 99.7 kg·ha^−1^, was associated with lower main-vine length during crop development, although final main-vine length at 144 DAT did not differ significantly from the control. This strategy also shifted the first visible flower bud to higher nodes and reduced marketable fruit number and yield without improving fruit quality. Fruit quality was influenced more consistently by harvest date than by GB strategy.

These findings are limited to one cultivar, one commercial greenhouse, and one production season and therefore should not be generalized beyond comparable production conditions. They do not exclude a protective role for GB under more severe or sustained low-temperature stress, but they indicate that repeated GB application requires crop- and environment-specific validation. Because cumulative GB input covaried with application frequency and the commercial formulation contained 11% total N, the present design cannot distinguish the effects of GB exposure and application frequency from any nutritional contribution associated with formulation-associated N. Future studies should therefore manipulate GB concentration and application frequency independently and include an appropriate N-matched or formulation control. Routine calendar-based application of approximately 11.7 g·L^−1^ GB (~100 mM) cannot be recommended for cool-season passion fruit production under conditions comparable to those of this study.

## Figures and Tables

**Table 1:** Greenhouse temperature metrics during three specified observational windows: winter summary, GB treatment, and flowering observation periods ^i^.

Temperature Metric ^ii^	Value
Mean daily maximum temperature from December to February ^iii^	29.5 °C
Mean daily minimum temperature from December to February	12.9 °C
Mean daily temperature range from December to February	16.6 °C
Minimum recorded temperature during the treatment period	7.0 °C
Number of days with a daily minimum temperature below 10 °C ^iv^	12 days
Cumulative exposure below 10 °C ^v^	59 h
Cumulative daytime exposure below 10 °C	10 h
Cumulative nighttime exposure below 10 °C	49 h
Proportion of total exposure below 10 °C occurring at night	83.1%
Mean daytime temperature during the flowering observation period	22.7 °C
Mean nighttime temperature during the flowering observation period	15.0 °C
Mean 24 h temperature during the flowering observation period	18.6 °C

^i^ The GB treatment period was from 30 October 2025 to 5 March 2026; the winter summary period was from 1 December 2025 to 28 February 2026; and the flowering observation period was 56.5–130.0 days after treatment (25 December 2025 to 9 March 2026). ^ii^ Air temperature was recorded hourly at hh:25 using a HOBO MX2301A temperature and relative humidity data logger positioned near the center of the greenhouse. ^iii^ Daily maximum, minimum, and mean temperatures were calculated from all hourly records within each calendar day. Daily temperature range was calculated as daily maximum minus daily minimum. ^iv^ A day with a minimum temperature below 10 °C was defined as a calendar day containing at least one hourly observation below 10 °C. ^v^ Cumulative exposure was the count of hourly observations below 10 °C; 10 °C was a descriptive acute-exposure index, not a verified flowering threshold. Day/night classification was based on local sunrise and sunset.

**Table 2:** Effects of cumulative glycine betaine (GB) application rate on the onset of visible reproductive development in ‘Tainung No. 1’ passion fruit.

Cumulative GB Application Rate (kg·ha^−1^)	Node Position of First Visible Flower Bud				Days to First Visible Flower Bud (DAT)				Days to First Flower Opening (DAT)		
0	20.8	±	1.5 ^i^	b ^ii^	21.0	±	2.2	ab	56.8	±	2.5
42.5	19.9	±	1.5	b	19.6	±	2.1	b	56.5	±	2.5
59.8	21.1	±	1.5	ab	21.7	±	2.2	ab	57.5	±	2.6
99.7	27.1	±	1.7	a	29.9	±	2.7	a	62.6	±	2.7
*p*-value	0.015				0.023				0.245		

^i^ Data represent means ± SE (*n* = 5). ^ii^ Within each column, means followed by the same letters or no letter are not significantly different (Tukey’s HSD test, *p* < 0.05). DAT = days after treatment.

**Table 3:** Effects of cumulative glycine betaine (GB) application strategy and harvest date on fruit dimensions, fruit mass, and peel CIE color of ‘Tainung No. 1’ passion fruit.

Days After Treatment (DAT) ^i^	Cumulative GB Application Rate (kg·ha^−1^)	Fruit Length (mm)			Fruit Diameter (mm)			Fruit Mass (g·fruit^−1^)				L* ^iii^				a*			b*			
187	0.0	64.3	±	1.1 ^ii^	57.1	±	1.0	59.0	±	2.5		28.5	±	1.0	abc ^iv^	20.7	±	1.4	8.1	±	1.2	
	42.5	62.5	±	2.5	56.3	±	2.3	54.5	±	5.5		33.0	±	2.4	ab	27.5	±	4.3	12.1	±	4.0	
	59.8	64.5	±	1.8	56.2	±	1.6	57.0	±	4.0		34.1	±	1.8	a	23.9	±	2.6	10.3	±	2.4	
	99.7	67.3	±	2.7	60.3	±	2.5	55.8	±	6.7		25.0	±	1.8	abcd	18.9	±	3.0	5.0	±	1.6	
194	0.0	64.3	±	1.1	57.0	±	1.0	60.4	±	2.5		25.0	±	0.9	bcd	18.0	±	1.2	4.7	±	0.7	
	42.5	66.6	±	1.1	59.2	±	1.0	65.8	±	2.7		25.4	±	0.9	bcd	20.5	±	1.4	5.4	±	0.8	
	59.8	66.2	±	1.1	59.2	±	1.0	63.8	±	2.7		24.7	±	0.8	cd	18.7	±	1.2	4.5	±	0.7	
	99.7	64.4	±	1.1	57.7	±	1.0	63.7	±	2.7		24.0	±	0.8	d	19.3	±	1.3	4.7	±	0.7	
200	0.0	67.4	±	1.1	57.8	±	1.0	70.6	±	2.9		25.7	±	0.9	bcd	20.3	±	1.3	6.2	±	0.9	
	42.5	65.3	±	1.1	58.5	±	1.0	70.8	±	3.0		25.2	±	0.9	cd	19.3	±	1.3	5.3	±	0.8	
	59.8	68.2	±	1.1	59.9	±	1.0	73.4	±	3.1		23.9	±	0.8	d	17.4	±	1.1	4.4	±	0.6	
	99.7	66.5	±	1.1	59.0	±	1.0	70.1	±	2.9		25.4	±	0.9	bcd	19.9	±	1.3	5.8	±	0.8	
		Pooled data																				
187		64.7	±	2.0	57.5	±	1.9	56.6	±	4.7	c	30.2	±	1.7	a	22.8	±	2.8	8.9	±	2.3	a
194		65.4	±	1.1	58.3	±	1.0	63.4	±	2.6	b	24.8	±	0.9	b	19.1	±	1.3	4.9	±	0.7	b
200		66.8	±	1.1	58.8	±	1.0	71.2	±	3.0	a	25.0	±	0.9	b	19.2	±	1.3	5.4	±	0.8	b
	0.0	65.3	±	1.1	57.3	±	1.0	63.4	±	2.6		26.4	±	0.9		19.7	±	1.3	6.4	±	0.9	
	42.5	64.8	±	1.6	58.0	±	1.5	63.7	±	3.7		27.9	±	1.4		22.4	±	2.3	7.6	±	1.8	
	59.8	66.3	±	1.4	58.5	±	1.2	64.7	±	3.2		27.6	±	1.2		20.0	±	1.7	6.4	±	1.2	
	99.7	66.1	±	1.6	59.0	±	1.5	63.2	±	4.1		24.8	±	1.2		19.4	±	1.9	5.1	±	1.1	
		*p*-value																				
DAT		0.110			0.437			<0.001				<0.001				0.064			0.003			
GB		0.565			0.381			0.946				0.111				0.366			0.462			
GB × DAT		0.417			0.604			0.824				0.009				0.297			0.276			

^i^ DAT, GB, and DAT × GB indicate the effects of days after treatment, glycine betaine treatment, and their interaction, respectively. ^ii^ Data are means ± SE (*n* = 5). ^iii^ L*, a*, and b* are CIE color coordinates, where L* represents lightness, a* represents the green (−) to red (+) axis, and b* represents the blue (−) to yellow (+) axis. ^iv^ Within each column, means followed by the same lowercase letter or no letter are not significantly different (*p* < 0.05, Tukey’s HSD test).

**Table 4:** Effects of cumulative glycine betaine (GB) application strategy and harvest date on soluble solids content (SSC), titratable acidity (TA), SSC/TA ratio, and juice content of ‘Tainung No. 1’ passion fruit.

Days After Treatment (DAT) ^i^	Cumulative GB Application Rate (kg·ha^−1^)	SSC (°Brix)				TA (%) ^iv^				SSC/TA				Juice Content (%)		
187	0.0	18.6	±	0.3 ^ii^	b ^iii^	2.42	±	0.10		7.83	±	0.32	abc	42.8	±	0.5
	42.5	18.1	±	0.6	ab	3.05	±	0.32		5.97	±	0.57	c	38.8	±	1.2
	59.8	18.9	±	0.4	ab	2.70	±	0.19		7.00	±	0.47	bc	39.9	±	0.8
	99.7	18.8	±	0.6	ab	2.16	±	0.20		8.77	±	0.84	abc	43.8	±	1.2
194	0.0	20.0	±	0.3	a	2.22	±	0.09		9.29	±	0.39	a	43.0	±	0.8
	42.5	18.3	±	0.3	b	2.22	±	0.09		8.27	±	0.34	abc	42.7	±	0.8
	59.8	18.6	±	0.3	ab	2.44	±	0.10		7.71	±	0.32	abc	42.9	±	0.8
	99.7	18.5	±	0.3	ab	2.44	±	0.10		7.60	±	0.32	bc	40.6	±	0.8
200	0.0	18.1	±	0.3	b	2.14	±	0.09		8.78	±	0.36	ab	45.7	±	0.9
	42.5	18.4	±	0.3	b	2.07	±	0.08		8.95	±	0.37	ab	42.7	±	0.8
	59.8	18.2	±	0.3	b	2.06	±	0.08		8.97	±	0.37	ab	42.6	±	0.8
	99.7	18.7	±	0.3	ab	2.06	±	0.08		9.04	±	0.37	ab	42.1	±	0.8
		Pooled data														
187		18.6	±	0.5	ab	2.58	±	0.20	a	7.39	±	0.55	a	41.3	±	0.9
194		18.9	±	0.3	a	2.33	±	0.10	a	8.22	±	0.34	b	42.3	±	0.8
200		18.3	±	0.3	b	2.08	±	0.08	b	8.93	±	0.37	c	43.3	±	0.8
	0.0	18.9	±	0.3		2.26	±	0.09		8.63	±	0.36		43.8	±	0.7
	42.5	18.3	±	0.4		2.45	±	0.16		7.73	±	0.43		41.4	±	1.0
	59.8	18.6	±	0.3		2.40	±	0.13		7.89	±	0.39		41.8	±	0.8
	99.7	18.7	±	0.4		2.22	±	0.13		8.47	±	0.51		42.2	±	0.9
		*p*-value														
DAT		0.019				<0.001				0.001				0.015		
GB		0.392				0.265				0.042				0.072		
GB × DAT		0.003				0.058				0.013				0.195		

^i^ DAT, GB, and DAT × GB indicate the effects of days after treatment, glycine betaine treatment, and their interaction, respectively. ^ii^ Data are means ± SE (*n* = 5). ^iii^ Within each column, means followed by the same lowercase letter or no letter are not significantly different (*p* < 0.05, Tukey’s HSD test). ^iv^ TA was expressed as % citric acid equivalents.

**Table 5:** Foliar treatment program and cumulative glycine betaine (GB) and nitrogen (N) inputs in ‘Tainung No. 1’ passion fruit ^i^.

GB Solution Concentration (g·L^–1^) ^ii^	Before Trellising (≤2 m)			After Trellising (≥2 m)			Cumulative Application Rate (kg·ha^–1^)	Cumulative N Applied with GB Formulation (kg·ha^–1^)
	Number of Applications	Spray Volume per Application (L·ha^–1^)	GB Input During Stage (kg·ha^–1^)	Number of Applications	Spray Volume per Application (L·ha^–1^)	GB Input During Stage (kg·ha^–1^)		
0	6	148.2	0.0	13	666.8	0.0	0.0	0
11.7	2		3.5	5		39.0	42.5	4.8
	3		5.2	7		54.6	59.8	6.8
	8		13.9	11		85.8	99.7	11.3

^i^ The experiment was conducted from 30 October 2025 to 5 March 2026, spanning 18 weeks and including 19 weekly application dates. ^ii^ The control received water plus 0.02% (*v*/*v*) surfactant at the corresponding control treatment events. GB-containing treatments were prepared at the same active-ingredient concentration; cumulative input was generated by differences in application frequency and canopy-dependent spray volume. The formulation contained 97% GB and 11% total N by product mass.

**Figure A1:**
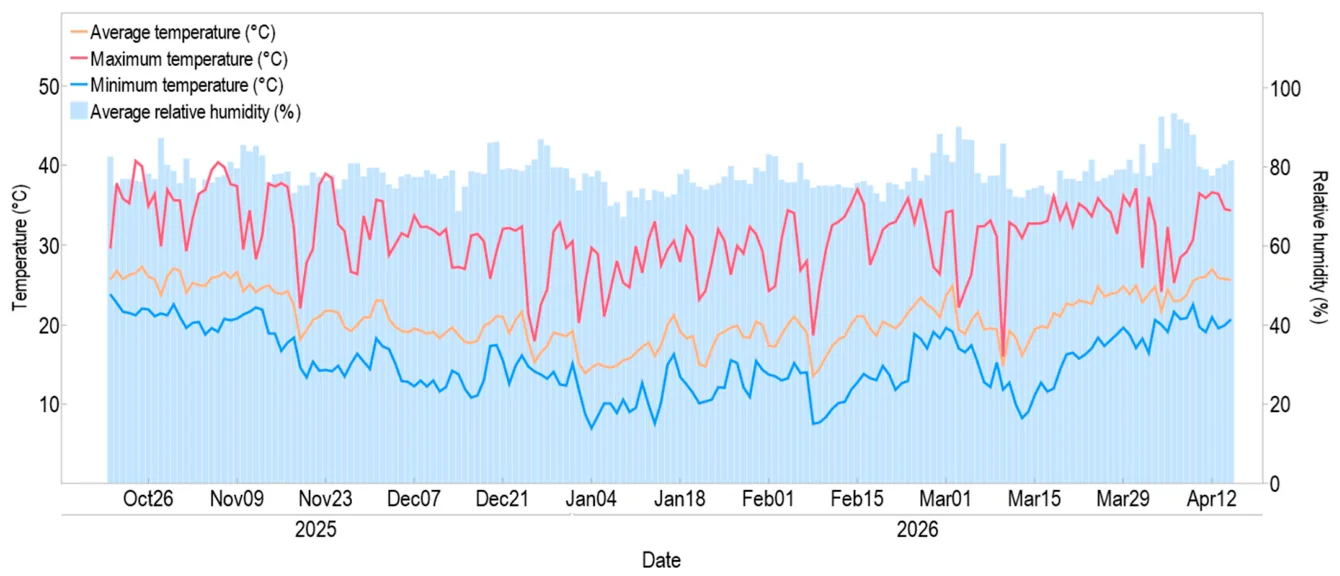
Daily changes in maximum, average, and minimum air temperatures, and average relative humidity inside the greenhouse during the experimental period (20 October 2025 to 15 April 2026).

## Data Availability

The raw data supporting the conclusions of this article will be made available by the authors on request.

## Supplementary Materials

The following supporting information can be downloaded at https://www.mdpi.com/article/10.3390/plants15213455/s1, Table S1: Summary of TAPMC grading criteria ^i^ for improved passion fruit used to define marketability in the present study. Reference [31] is cited in the Supplementary Materials.

## Appendix A. Greenhouse Environmental Conditions and Glycine Betaine Application Schedule

Greenhouse temperature and relative humidity were recorded from 20 October 2025 to 15 April 2026 (Figure A1). From October to mid-November 2025, the daily mean temperature generally remained above 20 °C. However, the daily maximum temperature reached 40 °C on some days, whereas the daily minimum temperature ranged from 18 to 21 °C, indicating pronounced diurnal temperature fluctuations during this period. Temperatures gradually declined after mid-November. On 19 November, the daily mean temperature decreased to 18.1 °C, and the daily maximum temperature was only 22.1 °C.

Temperatures in December were generally lower than those in November, with smaller fluctuations in daily maximum and minimum temperatures. During a cold event in late December, the daily mean temperature in the greenhouse decreased to 15.3 °C on 26 December and then gradually increased beginning on 29 December. Temperatures remained relatively low during the first half of January 2026. From 1 to 14 January, daily mean temperatures ranged from 14.6 to 17.7 °C, and the daily minimum temperature decreased to as low as 7.0 °C. Another period of low temperatures occurred from 19 to 24 January, during which daily mean temperatures ranged from 14.7 to 18.7 °C.

Temperatures remained relatively low from early to mid-February. On 8 February, the daily mean, maximum, and minimum temperatures were 13.5, 18.7, and 7.6 °C, respectively. Temperatures gradually increased after 12 February, with daily means generally ranging from 18.5 to 24.9 °C. In early March, temperatures remained near 20 °C. Another cold event occurred on 13 March, when the daily mean and minimum temperatures were 16.2 and 8.3 °C, respectively. From late March through April, temperatures gradually increased, and daily mean temperatures generally remained above 20 °C.

Overall, low-temperature conditions during the experimental period occurred primarily from December 2025 through February 2026, followed by a gradual increase in greenhouse temperature after late March. Mean relative humidity in the greenhouse generally remained above 70% throughout the experimental period.

## References

[1] Yockteng R., Coppens d’Eeckenbrugge G., Souza-Chies T.T., Kole C. (2011). Passiflora. Wild Crop Relatives: Genomic and Breeding Resources.

[2] Rodriguez Castillo N., Ambachew D., Melgarejo L.M., Blair M.W. (2020). Morphological and agronomic variability among cultivars, landraces, and genebank accessions of purple passion fruit, *Passiflora edulis* f. *edulis*. HortScience.

[3] Lee W.-H., Chen S.-J. (2020). Spring early-harvest passion fruit production in the Kaohsiung–Pingtung region. Kaohsiung Dist. Agric. Newsl..

[4] Lee W.-H. (2021). Technological development enables spring passion fruit production in Taiwan. Kaohsiung Dist. Agric. Mon..

[5] Lim C.K., Oh S., Koh S.C. (2018). Shoot growth, flowering, and fruit development of passion fruit *(Passiflora edulis* var. *edulis* Sims.) in response to cold-temperature cut-off. Hortic. Sci. Technol..

[6] Chang Y.-S., Cheng C.-Y. (1988). On the flowering habits of passion fruits. J. Chin. Soc. Hortic. Sci..

[7] Chang Y.-S., Cheng C.-Y. (1992). Effects of temperature and light on growth and flower formation of passionfruit. J. Chin. Soc. Hortic. Sci..

[8] Liu F.-Y., Peng Y.-L., Chang Y.-S. (2015). Effects of temperature and ethylene response inhibitors on growth and flowering of passion fruit. Korean J. Hortic. Sci. Technol..

[9] Nave N., Katz E., Chayut N., Gazit S., Samach A. (2010). Flower development in the passion fruit *Passiflora edulis* requires a photoperiod-induced systemic graft-transmissible signal. Plant Cell Environ..

[10] Theocharis A., Clément C., Barka E.A. (2012). Physiological and molecular changes in plants grown at low temperatures. Planta.

[11] du Jardin P. (2015). Plant biostimulants: Definition, concept, main categories and regulation. Sci. Hortic..

[12] Chen T.H.H., Murata N. (2011). Glycine betaine protects plants against abiotic stress: Mechanisms and biotechnological applications. Plant Cell Environ..

[13] Annunziata M.G., Ciarmiello L.F., Woodrow P., Dell’Aversana E., Carillo P. (2019). Spatial and temporal profile of glycine betaine accumulation in plants under abiotic stresses. Front. Plant Sci..

[14] Dai T., Ban S., Han L., Li L., Zhang Y., Zhang Y., Zhu W. (2024). Effects of exogenous glycine betaine on growth and development of tomato seedlings under cold stress. Front. Plant Sci..

[15] Li N., Pu K., Ding D., Yang Y., Niu T., Li J., Xie J. (2023). Foliar spraying of glycine betaine alleviated growth inhibition, photoinhibition, and oxidative stress in pepper (*Capsicum annuum* L.) seedlings under low temperatures combined with low light. Plants.

[16] Ashraf M., Foolad M.R. (2007). Roles of glycine betaine and proline in improving plant abiotic stress resistance. Environ. Expt. Bot..

[17] Khalil L., AlTurki S.M., Sebaaly Z., Sajyan T.K., Sassine Y.N. (2020). A trial to enhance cold tolerance of passion fruit (*Passiflora edulis*) crop in Lebanon through foliar application of the natural osmoprotectant glycine betaine. Acta Hortic..

[18] Oliveira L.D.A., Nguyen N.T.T., Habibi N., Dabirimirhosseinloo M., Terada N., Sanada A., Koshio K. (2025). Exogenous application of glycine betaine to *Passiflora edulis* Sims f. *flavicarpa* to mitigate drought stress on two propagation methods. Int. J. Mol. Sci..

[19] Park E.-J., Jeknic Z., Chen T.H.H. (2006). Exogenous application of glycinebetaine increases chilling tolerance in tomato plants. Plant Cell Physiol..

[20] Li J., Van Gerrewey T., Geelen D. (2022). A meta-analysis of biostimulant yield effectiveness in field trials. Front. Plant Sci..

[21] Niu T., Zhang J., Li J., Gao X., Ma H., Gao Y., Chang Y., Xie J. (2023). Effects of exogenous glycine betaine and cycloleucine on photosynthetic capacity, amino acid composition, and hormone metabolism in *Solanum melongena* L. Sci. Rep..

[22] Mäkelä P., Peltonen-Sainio P., Jokinen K., Pehu E., Setälä H., Hinkkanen R., Somersalo S. (1996). Uptake and translocation of foliar-applied glycinebetaine in crop plants. Plant Sci..

[23] Mickelbart M.V., Chapman P., Collier-Christian L. (2006). Endogenous levels and exogenous application of glycinebetaine to grapevines. Sci. Hortic..

[24] Peng S., Garcia F.V., Laza R.C., Sanico A.L., Visperas R.M., Cassman K.G. (1996). Increased N-use efficiency using a chlorophyll meter on high-yielding irrigated rice. Field Crops Res..

[25] Zhang R., Yang P., Liu S., Wang C., Liu J. (2022). Evaluation of the methods for estimating leaf chlorophyll content with SPAD chlorophyll meters. Remote Sens..

[26] Ghosh A., Dey K., Bauri F.K., Dey A.N. (2017). Physico-chemical changes during fruit growth and developmental stages in yellow type passion fruit (*Passiflora edulis* f. *flavicarpa* Degener) accessions. J. Appl. Nat. Sci..

[27] Angami T., Kalita H., Baruah S., Ronya T., Bam B., Shukla K.K. (2017). Dynamics of physico-biochemical changes in passion fruit varieties across maturity. Int. J. Chem. Stud..

[28] Md Nor S., Ding P., Abas F., Mediani A. (2022). ^1^H NMR reveals dynamic changes of primary metabolites in purple passion fruit (*Passiflora edulis* Sims) juice during maturation and ripening. Agriculture.

[29] Tang Y., Wang P., Chen J., Li L., Tang L., Huang W., Wei X., Xu J. (2025). The accumulation pattern of sugars and organic acids in diverse fruit pulps coordinates sweet–sour taste of passion fruit. LWT.

[30] Afonso S., Oliveira I., Guedes F., Meyer A.S., Gonçalves B. (2024). Glycine betaine and seaweed-based biostimulants improved leaf water status and enhanced photosynthetic activity in sweet cherry trees. Front. Plant Sci..

[31] Taipei Agricultural Products Marketing Co., Ltd. (TAPMC) (2026). Grading and Packaging Standards: Passion Fruit (Improved Type). https://www.tapmc.com.tw/Pages/Market/Packing/G000C2.

