# Supplementary material for: Repeated Foliar Glycine Betaine Applications in Cool-Season Greenhouse Passion Fruit: Effects of Application Frequency on Growth and Marketable Yield
Source: Plants (Basel). 2026 Nov 5;15(21):3455. doi: 10.3390/plants15213455 (PMC13766139; doi:10.3390/plants15213455)
Supplement: Supplementary file 1 [file plants-15-03455-s001.zip › plants-4657420-supplementary.pdf]

Table S1. Summary of TAPMC grading criteria[i] for improved passion fruit used to define marketability in the present study.

Criterion	TAPMC category or description	Application in this study
Individual fruit mass	Large: ≥80 g; Medium: 60–79 g; Small: <60 g	All three size classes could be marketable; no minimum fruit-mass threshold was imposed
Extra grade	Same cultivar; appropriate maturity; complete fruit shape; superior coloration; smooth fruit surface; no visible defects	Marketable
First grade	Same cultivar; generally appropriate maturity; generally complete fruit shape; good coloration; relatively smooth surface; minor external defects permitted	Marketable
Standard grade	Quality below first grade but still possessing commercial value	Marketable
External defects	Includes defects caused by disease, insects, physiological disorders, or mechanical injury, such as decay, scars, water injury, cracking, abrasion, or compression injury	Fruit lacking commercial value because of such defects were classified as unmarketable

[i]Grading criteria were summarized from Taipei Agricultural Products Marketing Co., Ltd. (TAPMC) [31]. TAPMC size classes are grading categories and do not constitute a minimum fruit-mass requirement for marketability.
